# Clinical efficacy and cost-effectiveness of bespoke compared with over-the-counter foot orthoses for plantar heel pain

**DOI:** 10.1186/1757-1146-3-S1-O22

**Published:** 2010-12-20

**Authors:** Katie Ring, Simon Otter

**Affiliations:** 1 University of Brighton, Brighton, UK

## Objectives and relationship to conference themes

Plantar heel pain is a common foot complaint often managed with orthoses; however, bespoke foot orthoses can be time-consuming and expensive to manufacture. Therefore we aimed to compare the clinical efficacy and cost-effectiveness of bespoke foot orthoses and pre-fabricated foot orthoses for heel pain.

## Presentation topic

A total of 67 patients with plantar heel pain were recruited for this study; if, following initial assessment, foot orthoses were indicated patients were randomised to receive either bespoke orthoses or pre-fabricated semi-rigid orthoses (Powerstep). Clinical efficacy was assessed at 4 and 8 weeks using the Manchester foot pain and disability questionnaire and cost-effectiveness was determined by analysing the material, manufacture and labour costs.

## Participant outcomes

At baseline there were no appreciable differences in the two groups of patients in demographic or clinical parameters. At 4 and 8 weeks post intervention both types of orthoses had significantly reduced pain and disability (p <0.0001). The Powerstep insoles cost £17.45 (19.36 Euros) in total where as the bespoke orthoses were considerably more expensive at £26.86 (29.80 Euros) to provide.

## Relevance

For most patients with plantar heel pain pre-fabricated semi-rigid insoles such as the Powerstep devices used in this trial provide equal benefit to bespoke, casted foot orthoses, but are considerably cheaper.

